# *Helicobacter pylori* recombinant UreG protein: cloning, expression, and assessment of its seroreactivity

**DOI:** 10.1186/1756-0500-7-809

**Published:** 2014-11-18

**Authors:** Akbar Khalilpour, Sabariah Osman, Muhammad Hafiznur Yunus, Amutha Santhanam, Nagarajan Vellasamy, Rahmah Noordin

**Affiliations:** Institute for Research in Molecular Medicine (INFORMM), Universiti Sains Malaysia, 11800, Minden, Penang, Malaysia; Hospital Seberang Jaya, Penang, Malaysia

**Keywords:** *Helicobacter pylori*, UreG, Cloning, Expression, Recombinant protein, Seroreactivity

## Abstract

**Background:**

*Helicobacter pylori* is a human pathogen and during the process of infection, antigens from the bacterium elicit strong host humoral immune responses. In our previous report, native *H. pylori* UreG protein showed good reactivity with sera from *H. pylori* patients. This study was aimed at producing the recombinant form of the protein (rUreG) and determining its seroreactivities.

**Methods:**

The coding sequence of *H. pylori* UreG was cloned and the recombinant protein expressed and purified by affinity chromatography using nickel nitrilotriacetic acid (Ni-NTA) resin. The antigenicity of rUreG to detect *H. pylori* specific antibodies was determined by western blot, using HRP-conjugated anti-human IgG and IgA antibodies as probes. A total of 70 sera, comprising 30 positive and 40 control serum samples, were used. The positive sera were from culture-positive *H. pylori*-infected patients with duodenal ulcers, gastric ulcers, or gastritis. The control sera comprised three types of samples without detectable *H. pylori* antibodies, i.e. healthy individuals (with no history of gastric disorders) (n = 10); patients who attended an endoscopy clinic (because of gastrointestinal complaints) but were *H. pylori* culture negative (n = 20); and people with other diseases (n = 10). Additionally, hyperimmune mice serum against rUreG was raised and tested with the native and recombinant UreG protein.

**Results:**

The *ureG* gene fragment was successfully cloned and expressed in both soluble and insoluble forms. Western blots on rUreG protein showed 70% (21/30) and 60% (18/30) reactivity with patients’ sera when probed with HRP-conjugated anti-human IgG and IgA antibodies, respectively; and the combination of the IgG and IgA western blots showed reactivity of 83.3% (25/30). By comparison, 97.5% and 92.5% of the control sera showed no reactivity when probed with HRP-conjugated anti-human IgG and IgA antibodies, respectively. Both the *H. pylori* lysate antigen and rUreG protein displayed a distinctive band at the expected molecular weight when probed with the hyperimmune mice serum.

**Conclusion:**

The rUreG protein was successfully cloned and expressed and showed good reactivity with *H. pylori* culture-positive patients’ sera and no reactivity with most control sera. Thus, the diagnostic potential of this recombinant protein merits further investigation.

## Background

*Helicobacter pylori* is a helical- or spiral-shaped, microaerophilic, Gram-negative bacterium that colonizes the apical side of human gastric epithelial cells and mucous layers [1]. The discovery and successful culture of *H. pylori* by Marshall and Warren in 1982 revolutionized the diagnosis and treatment of gastroduodenal disease [2] *.* This organism has been categorized as a class I carcinogen by the World Health Organization [3, 4] and direct evidence of carcinogenesis was recently demonstrated in an animal model. *H. pylori* infection is associated with peptic ulcer diseases such as gastric or duodenal ulcer, atrophic gastritis, gastric cancer, and mucosa-associated lymphoid tissue lymphoma [2, 5]. Hence, the identification and prevention and/or treatment of *H. pylori* infection can prevent considerable mortality resulting from chronic infection. During the process of *H. pylori* infection, the antigens secreted from the bacterium elicit strong antibody responses in the host; thus, secreted *H. pylori* antigens may be studied as potential infection biomarkers [6]. Various *H. pylori* proteins had been employed as infection markers for diagnosis, such as CagA, VacA, HspB, FlaA, FlaB, and UreC [7, 8].

On initial infection of gastric cells, the host responds strongly to the presence of *H. pylori* through both the innate and acquired immune systems. Antigen-presenting B cells differentiate into plasma cells, which produce antibodies such as IgA, IgG, and IgM as the second line of defense, with IgA as the first antibody secreted [9, 10]. During the early stages of *H. pylori* infection, IgM antibodies are secreted by plasma cells before there is a sufficient titer of IgG in the blood [6]. Human studies have shown that the presence of anti-*H. pylori* IgA in maternal milk is associated with delayed colonization in infants, suggesting that IgA can block infection. In another study by Argent and Atherton (2007), IgG antibodies proved to be a useful tool for the serodiagnosis of *H. pylori* infection [11]. In summary, these studies suggested that anti-*H. pylori* antibodies can be used for diagnosis, to reduce infection and, by inference, could be an effector mechanism in prophylactic vaccination as well. During *H. pylori* infection, antibodies of the classes IgG and IgA can be detected, while IgM antibodies have been found to have little diagnostic utility. This may be explained by the fact that *H. pylori* infection is a chronic condition and IgM antibody is secreted in the acute phase of infection [12].

One approach to identify antigen candidates for diagnostic purposes is to isolate the bacterium from the biopsy samples of patients and use the antisera from the patients to screen for reactive proteins in western blot analysis. This would then be followed by purification of the immunoreactive proteins, determination of the amino acid sequence, nucleotide sequence elucidation, cloning, and recombinant protein expression [13].

Using a similar approach, Khalilpour *et al.* (2013) studied a local (Malaysian) *H. pylori* isolate to identify potential diagnostic markers. Four protein bands were identified by western blot analysis using IgG from serum samples of culture-positive patients and control sera [14]. Out of the four proteins identified (UreG, UreB, CagI, and pyrroline-5-carboxylate dehydrogenase), UreG was selected for further work because it is a novel protein in terms of potential utility for diagnosis. It is also an interesting protein because it is frequently required to complete the biosynthesis of nickel enzymes [15]. In the present study, the UreG protein was investigated further. We report the cloning and expression of the UreG coding sequence and determination of its seroreactivity.

## Methods

### Ethical approval

Written informed consent from the patient was obtained for use of his duodenal biopsy sample in this study. In addition written informed consents were obtained from each patient prior to collection of their serum samples. Approval for this study was obtained from the Universiti Sains Malaysia Human Research Ethics Committee (Ref no: USMKK/PPP/JEPeM [214.3.4]). This approval included the collection and use of the *H. pylori* strain isolated from the human duodenal biopsy sample, the collection and use of serum samples and the use of stored control serum samples. The use of mice in this study was approved by Universiti Sains Malaysia Animal Research Ethics Committee [Ref. no: USM/Animal Ethics Approval/2012 (75) (407)].

### Bacterial culture and extraction of genomic DNA

The *H. pylori* strain used in this study (HpUSM3121108) was isolated from a human duodenal biopsy sample obtained from a patient with a duodenal ulcer at the Sebarang Jaya Hospital, Penang, Malaysia. *H. pylori* was cultured on tryptic soy blood agar with 5% defibrinated sheep blood under microaerophilic conditions (10% CO_2_, 5% oxygen, 85% nitrogen in air) at 37°C for 5–7 days [16, 17]. The bacterial cells were aseptically harvested by adding 3 mL phosphate-buffered saline (PBS, pH 7.2) to the agar plate; the bacterial suspension from the plate was then collected and placed in a 10-mL centrifuge tube, and centrifuged at 3000 × *g* at 4°C for 15 min. The pellet was washed by resuspending in 1 mL of PBS, transferred into a 1.5-mL microcentrifuge tube, followed by centrifugation at 11,000 × *g*. The washing step was repeated twice, then the final fresh bacterial cell pellet was used for genomic DNA extraction using the Bacterial Genomic DNA Extraction Kit (DNeasy; Qiagen, Hilden, Germany). The DNA was suspended in TE buffer and stored at -20°C [18, 19].

### DNA amplification

The sequence of the *ureG* gene was deposited in the GenBank database with accession number KC576840 [20]. The *ureG* gene was amplified by polymerase chain reaction (PCR) using a forward primer containing a *Hin*dIII restriction site and a reverse primer containing an *Eco*RI site: 5′-AAG CTT TCA ATC TTC CAA TAA AGC GTT G-3′ and 5′-GAA TTC ATG GTA AAA ATT GGA GTT TGT GG-3′. PCR was performed in an automatic thermal cycler using a 50-μL reaction mixture containing 7 μL of 10× PCR buffer, 3 μL of sample DNA, 1 μL of 10 mmol/L dNTPs, 2 μL of 20 pmol/L primers, and 2.5 U of Taq polymerase. The mixture was incubated for 2 min at 94°C for initial denaturation of the target DNA, then 35 cycles of denaturation at 94°C for 1 min, annealing at 53.8°C for 1 min, and elongation at 72°C for 2 min. The amplified products (5 μL) were analyzed by agarose electrophoresis and then visualized under ultraviolet light.

### Recombinant plasmid construction and purification

The PCR product was purified using the PCR Fragment Recovery Kit (Promega, Madison, WI, USA). In preparation for cloning, an A-tailing reaction was performed. The reaction mixture comprised 10 μL of purified PCR product, 1 μL of 10× PCR buffer (with MgCl_2_), 1 μL of 12.5 mmol/L of dATP, and 3.75 U of Taq polymerase. After incubation at 72°C for 30 min, the A-tailed PCR product was cloned in the pCR®2.1-TOPO vector supplied in the TOPO™-TA Cloning Kit (Invitrogen, Carlsbad, CA, USA). The reaction contained 4 μL of purified PCR product, 1 μL of salt solution, 1 μL of pCR®2.1-TOPO vector, and 1 μL of water (Bioline, USA). The reaction mixture was then transformed into TOP10F *Escherichia coli* and positive clones were selected on agar containing X-gal and identified by PCR and restriction enzyme digestion. A single positive bacterial colony was cultivated in 3 mL of LB broth containing 100 mg/L of ampicillin, with shaking (300 rpm), at 37°C overnight. Recombinant plasmids (TOPO®/*ureG*) were extracted using the DNA Purification Kit (Promega) and checked by PCR and restriction endonuclease digestion.

For sub-cloning into the pRSET vector (Invitrogen), endonuclease digestion was performed on the TOPO®/*ureG* recombinant plasmids and pRSET version “a” (Invitrogen) to generate fragments with *Eco*RI and *Hin*dIII digestion sites. Then, a ligation reaction was prepared, which consisted of 17 μL of gel-purified insert, 1 μL of pRSET vector, 2 μL of DNA dilution buffer, and 10 μL of rapid ligation buffer. The reaction was started by adding 5 U of T4 DNA ligase, the mixture was mixed by pipetting and then incubated at 15–25°C for 5 min. The circular recombinant plasmid was then transformed into a competent expression host [*E. coli* BL21 (DE3) pLysS] (Invitrogen) and plated onto LB agar which contained 100 μg/mL of ampicillin and 35 μg/mL of chloramphenicol. DNA sequencing of the cloned and sub-cloned *ureG* gene of the recombinant plasmid was performed by the Centre for Chemical Biology (CCB) at Universiti Sains Malaysia. The nucleotide and amino acid sequences were then analyzed using the BioEdit software (Ibis Biosciences, Carlsbad, USA) and compared with published sequences in the public databases.

### Expression of recombinant protein

A pre-culture was first established by inoculating a single colony of the recombinant bacteria from a freshly subcultured LB plate (containing 100 μg/mL of ampicillin and 35 μg/mL of chloramphenicol) into 100 mL of Terrific broth (TB) containing the same antibiotics as above, followed by overnight incubation at 37°C, with shaking (180 rpm). The TB was prepared by dissolving 12 g bacto-tryptone, 24 g yeast extract, and 4 mL glycerol in 900 ml deionized water, then autoclaving. The TB was then made up to 1 L with salt solution prior to use. The salt solution was prepared by dissolving 125.4 g dipotassium hydrogen phosphate (K_2_HPo_4_) and 23.1 g potassium dihydrogen phosphate (KH_2_PO_4_) in 1 L deionized water, then autoclaving.

For large-scale expression, 4 L (eight 500-mL cultures in 1-L flasks) of fresh TB were used. Each flask was inoculated with 25 mL of the above pre-culture and incubated at 37°C, with shaking (200 rpm) until the OD_600_ reached 0.5. The recombinant bacteria were induced to express recombinant UreG (rUreG) by the addition of 1 mM isopropyl β-D-1-thiogalactopyranoside (IPTG; Fermentas, Hanover, MD, USA), followed by incubation at 37°C, with shaking (200 rpm), for 4.5 h. The cells were then harvested by centrifugation at 10,000 × g at 4°C for 10 min. The cell pellet was resuspended in 10 mL of lysis buffer (containing 10 mM imidazole and 0.1% Triton-X 100), lysozyme (0.5 mg/mL; Amresco, Solon, OH, USA), and a cocktail of protease inhibitors (14.8 μg/mL; Roche Applied Science, Indianapolis, IN, USA), incubated on ice for 30 min, followed by lysis using a French press 40 K (Thermo Spectronic, Rochester, NY, USA). The supernatant (containing soluble protein) and the pellet (containing inclusion bodies) were separated by centrifugation at 10,000 × *g* at 4°C for 10 min, and both components were retained for subsequent procedures.

### Purification of rUreG under native conditions

*DNase*I was added (5 μg/mL) to the supernatant (prepared as above), incubated on ice for 10–15 min, centrifuged at 10,000 × *g* at 4°C for 30 min, and then syringe filtered (0.45 μm). Finally, the supernatant was incubated with 1 mL of washed nickel nitrilotriacetic acid (Ni-NTA) resin (Qiagen) packed in a column for 1 h at 4°C. To maximize the purity of the recombinant protein, the affinity column was washed with buffers containing a gradient of increasing imidazole concentrations. This comprised four buffers, each containing the basic buffer (50 mM NaH_2_PO_4_, 300 mM NaCl) with the addition of imidazole concentrations of 10–20 mM, 30 mM, and 40 mM. Each washing step was performed with 10 column volumes of buffer. Finally, the purified recombinant protein was eluted with 10 column volumes of elution buffer (50 mM NaH_2_PO_4_, 300 mM NaCl, and 250 mM imidazole); this buffer contained an excess of imidazole to allow displacement of the His-tag from nickel co-ordination, thus the His-tagged recombinant protein was able to be eluted. The protein-containing fractions were pooled and buffer exchanged into PBS containing 1 M urea using a spin column with a 3-kDa molecular weight cut-off (Vivapsin; Sartorius, USA), this was repeated three times. The protein concentration was determined using a commercial protein assay kit (*RC DC™*; BioRad, Hercules, CA, USA), and the protein was stored at -80°C.

### Purification of rUreG under denaturing conditions

The protein pellet, prepared as described above, was dissolved in 15 mL of denaturing lysis/binding buffer (7 M urea, 100 mM NaH_2_PO_4_, 10 mM Tris-HCl, pH 8.0) and incubated in a shaker for 15–30 min, then centrifuged at 10,000 × *g* at 4°C for 10 min. *DNase*I was mixed with the supernatant and incubated on ice for 30 min, then centrifuged at 10,000 × *g* at 4°C for 30 min and syringe filtered (0.45 μm). The supernatant was then incubated with 1 mL of washed Ni-NTA resin (Qiagen) for 1 h at 4°C. The mixture was loaded into a purification column pre-equilibrated with denaturing lysis buffer. The column was washed twice with 10 mL of denaturing buffer (8 M urea, 100 mM NaH_2_PO_4_, 10 mM Tris-HCl, pH 6.1). The recombinant protein was eluted as 500-μL fractions with 10 mL of denaturing elution buffer D (8 M urea, 100 mM NaH_2_PO_4_, 10 mM Tris-Cl, pH 5.9) and the protein content was confirmed using a protein assay kit (Bio-Rad). More fractions were collected using elution buffer E (8 M urea, 100 mM NaH_2_PO_4_, 10 mM Tris-Cl, pH 4.5) until no more protein was observed. The purified fractions were pooled and buffer-exchanged five times (as described above) into storage buffer containing 50 mM Tris-HCl, 150 mM NaCl, and 5% glycerol. The protein concentration was determined using a commercial protein assay (*RC DC™*, BioRad), and the protein was stored at -80°C.

### Serum samples

The serum samples were first tested using a commercial *H. pylori* IgG-ELISA kit (Adaltis, Bologna, Italy) and divided into several categories on the basis of their serological profiles. Group I: serum samples from patients whose biopsy cultures were positive for *H. pylori* and anti-*H. pylori* IgG antibodies (n = 30). This group comprised two patients with duodenal ulcers, five patients with gastric ulcers, 20 patients with gastritis, and three patients with normal scope findings. Group II: serum samples from healthy individuals with no history of gastric disorders (n = 10). Group III: serum samples from patients with gastrointestinal complaints who were referred to the endoscopy unit; however, their tissue biopsy cultures were negative for *H. pylori* (n = 20). Group IV: serum samples from people with other diseases, such as typhoid, leptospirosis, *E. coli* septicemia, shigellosis, *Staphylococcus aureus* septicemia, and amoebic liver abscess (n = 10). Sera from groups II–IV tested negative for anti-*H. pylori* IgG antibodies and served as control sera. Control sera were previously stored samples from the serum bank at INFORMM (Universiti Sains Malaysia).

### SDS-PAGE and western blot analysis

The recombinant protein preparations were each loaded at 20 μg per well onto a 12% resolving gel, and SDS-PAGE was performed at 100 V. The gel was transferred onto a nitrocellulose membrane (0.45 μm; BioRad) using a semi-dry transblot (BioRad), then blocked with 5% alkali-soluble casein overnight at 4°C. The membrane was then washed four times with Tris-buffered saline containing 0.05% Tween 20 (TBS-T; 50 mM Tris, 150 mM NaCl, 0.05% Tween-20). For the detection of histidine-tagged protein, the membrane was incubated with anti-His-HRP (Novagen, Germany) (1:1000) for 1 h at room temperature. After a washing step, substrate development was performed using enhanced chemiluminescence blotting reagent (Roche Diagnostics, Mannheim, Germany) and Kodak films (Kodak, USA).

For western blotting using the serum samples, after transfer of the recombinant protein onto the nitrocellulose membrane, the membrane was washed four times with TBS-T, then blocked with 1% blocking solution (Roche Diagnostics) for 1 h at room temperature on a shaker. After one more wash, the membrane was cut into strips, then incubated at 4°C overnight with different groups of serum samples as the primary antibody (1:25 dilution in TBS). Following three washing steps, the strips were incubated with monoclonal mouse anti-human IgG-HRP (1:4000 in TBS; Invitrogen) or monoclonal mouse anti-human IgA-HRP (1:2000 in TBS) for 1 h, followed by washing with TBS-T. Substrate development was performed as described above.

### In-gel digestion of proteins and MS-MS analysis

The recombinant protein band was manually excised from the Coomassie blue-stained gel and destained by incubating with 200 μL of destaining solution for 30 min at 37°C with shaking. The gel slice was digested and extracted using the Agilent Protein In-gel Trypsin Digestion Kit (Agilent, USA). The sample was then cleaned up using ZipTipC18 pipette tips (Zip-Tip U-C18; Millipore, Bedford, MA, USA), eluted, and concentrated using a vacuum concentrator (Eppendorf, Westbury, NY, USA) until completely dry. The protein was then analyzed using MALDI-TOF-TOF 4800 (ABSCIEX, Foster City, CA, USA).

### Production of hyperimmune mice serum against UreG recombinant protein and antigenicity determination

Pre-immunized serum was obtained from four mice (aged 4–6 weeks) by bleeding from the tail, then pooled for use as control serum [21]. Next, a mixture containing 200 μL of Freund’s complete adjuvant (CFA) and 200 μL of 200 μg UreG recombinant antigen was subcutaneously injected into each of the four mice. Two booster injections were given at 2-week intervals, one using Freund’s incomplete adjuvant (IFA) mixed with the recombinant antigen and another using the recombinant antigen alone. Blood from each mouse was centrifuged to extract the serum sample. Then, serum samples from all four mice were pooled for use as the primary antibody.

*H. pylori* lysate antigen and the purified UreG recombinant protein were electrophoresed at 20 μg per well on a 12% SDS-PAGE, then transferred onto nitrocellulose membrane as explained above. The membrane was blocked, washed, cut into strips, and incubated with the pooled mice serum, then probed with monoclonal human anti-mouse IgG-HRP (Invitrogen). The strips were then washed four times with TBS-T and developed using chemiluminescence substrate as described above.

## Results

**Figure 1:**
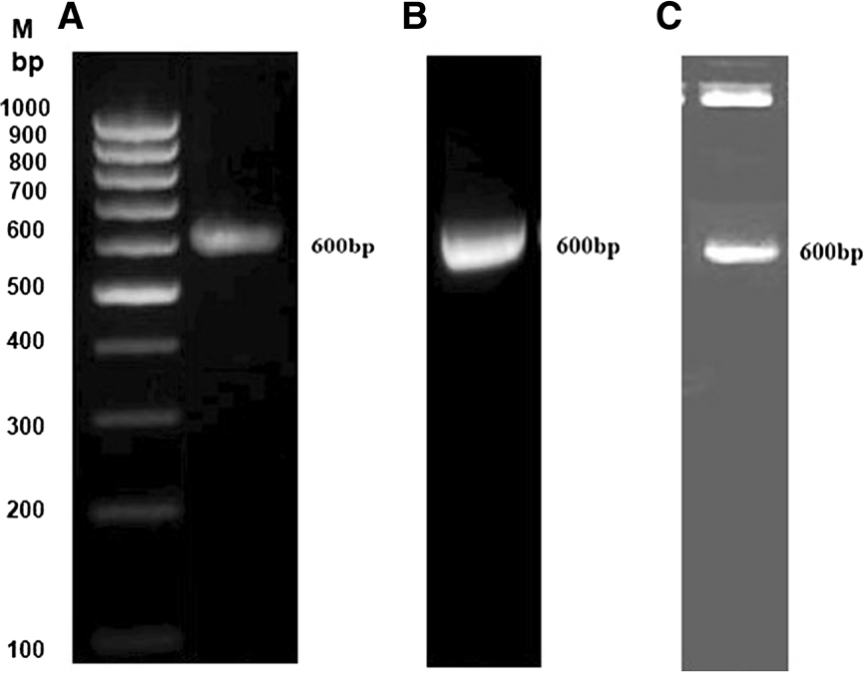
**PCR amplification of the** ***ureG*** **gene and verification of the recombinant prokaryotic expression plasmid pRSET-** ***ureG.*** **A**: PCR amplification of the *ureG* gene **B**: PCR screening of the pRSET/*ureG* recombinant **C**: Digestion product of pRSET/*ureG* with *Hin*dIII and *Eco*RI **M**: 100 bp DNA ladder.

The presence and orientation of the DNA sequences of the *ureG* gene in PCR2.1 TOPO and the pRSET constructs was confirmed by performing PCR with the vector and gene-specific primers and DNA sequencing. Figure 1 shows amplification of the *ureG* gene from the pRSET/*ureG* recombinant plasmid and excision of this gene from the plasmid using enzymes *Hin*dIII and *Eco*RI. DNA sequence analysis of the TOPO/*ureG* and pRSET/*ureG* constructs confirmed that *ureG* was in the correct reading frame (data not shown).

**Figure 2:**
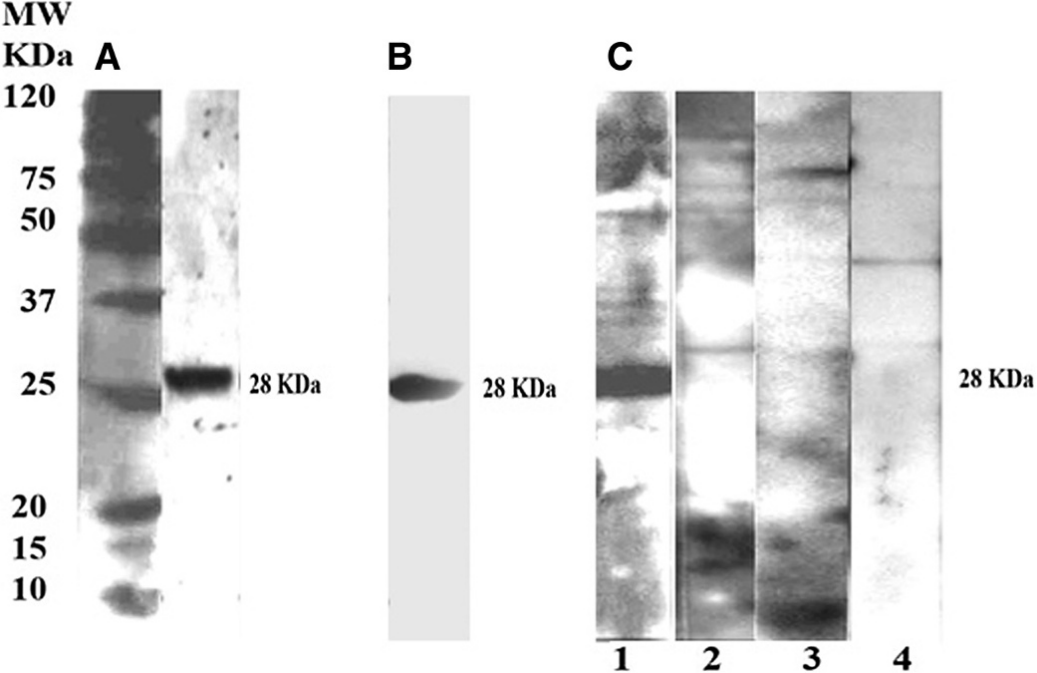
**Western blot analysis showing the immunogenicity and specificity of the purified UreG recombinant protein. A**: rUreG purified under native condition probed with monoclonal anti-histidine-HRP **B**: rUreG purified under denaturing condition probed with monoclonal anti-histidine-HRP **C**: rUreG incubated with primary antibodies from different groups of serum samples: 1: Primary antibody from the Group I serum sample (culture-positive patient) 2: Primary antibody from the Group II serum sample (healthy person) 3: Primary antibody from the Group III serum sample (culture-negative patients seronegative) 4: Primary antibody from the Group IV serum sample (patient with other disease) MW: low molecular weight marker.

Figure 2 shows the results of western blot analysis of the purified recombinant protein prepared from the supernatant and pellet of the lysed cells to check for the presence of the six-histidine fusion in the UreG recombinant protein; a thick distinctive band was observed at the expected molecular weight of approximately 28 kDa. MALDI TOF-TOF results confirmed the identity of the protein as UreG; with scores of 454 with 10 matched peptides and 462 with six matched peptides from bands excised from SDS-PAGE of native and denatured forms of the recombinant protein, respectively.

Although western blot analysis indicated that the expressed UreG target protein was present in both the supernatant and pellet of the lysed cells, the amount of protein in the pellet (insoluble protein) was 3 mg/L of culture, compared with 1.4 mg/L of culture (soluble protein) in the supernatant.

The UreG recombinant protein showed 70% (21/30) and 60% (18/30) reactivities with Group 1 sera (culture-positive patients) when probed with anti-human IgG-HRP and anti-human IgA-HRP respectively. When the results of the IgG and IgA western blots were combined, the positive reactivity rate was 83.3% (25/30). Among the control sera (Groups II, III, and IV), 97.5% and 92.5% were not reactive when probed with anti-human IgG-HRP and anti-human IgA-HRP, respectively.

**Figure 3:**
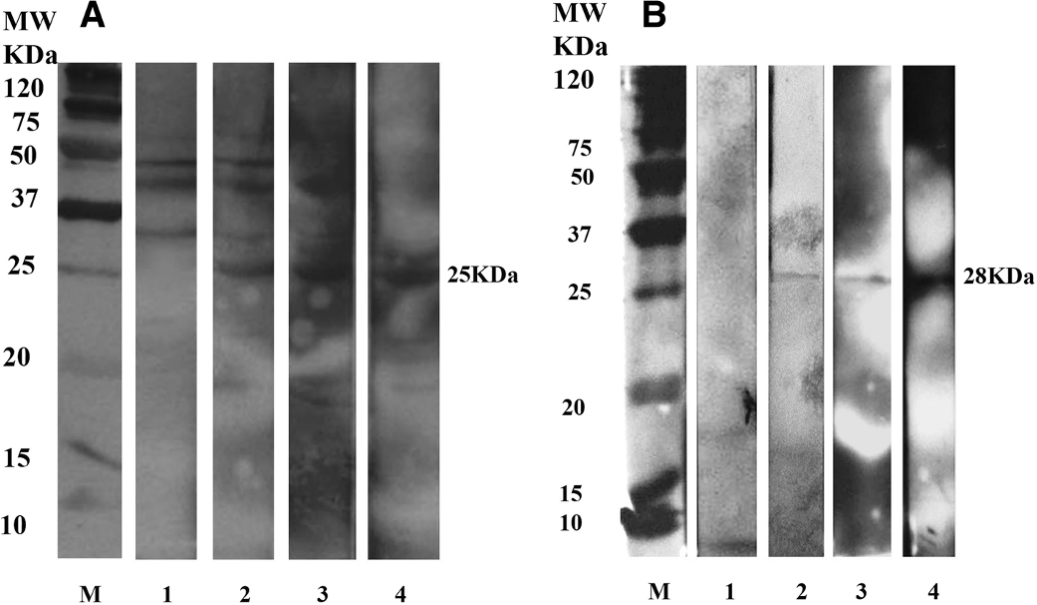
**Western IgG blot incubated with mice hyperimmune pooled serum. A**: native *H. pylori* antigen **B**: rUreG antigen 1: Pooled serum before 1st injection (control) 2: Pooled serum before 2nd injection 3: Pooled serum before 3rd injection 4: Pooled serum after 3rd injection MW: low molecular weight marker.

Both the *H. pylori* lysate antigen and purified UreG recombinant protein displayed a distinctive band at the expected molecular weights of 25 kDa and 28 kDa respectively when probed with hyperimmune mice serum (Figure 3). This demonstrated that the polyclonal antibody against UreG recombinant protein was recognized by both native and recombinant UreG proteins.

## Discussion

Several virulence-associated genes have been identified in *H. pylori* and these are believed to play a major role in the pathogenesis of this organism. The proteins expressed by some of these genes, such as UreB, VacA, CagA, HspB, FlaA, FlaB, and outer membrane proteins, have been investigated as diagnostic indicators of *H. pylori* infection [7, 8, 19, 22].

The production of a wide range of proteins was previously unattainable because of the limitations imposed by the use of native proteins. In recent times however, advances in recombinant technology have made it possible to produce a wide range of defined antigens in large quantities for use in diagnostic applications. For *H. pylori*, a variety of recombinant protein antigens, such as rUreB, rVacA, rCagA, rHpaA, rNapA, rFlaA, and rFlaB, have been constructed, expressed, and purified by affinity chromatography. These recombinant antigens could improve serodiagnosis of *H. pylori* infection, replacing the complex native *H. pylori* antigens [23–25]. In one study, rUreB was used as the antigen in ELISA to identify a specific antibody in serum samples from *H. pylori*-infected patients. In addition, the anti-rUreB rabbit polyclonal antibody was used to study UreB expression in different *H. pylori* isolates. A specific antibody against rUreB in the sera of *H. pylori*-infected patients and high frequencies of UreB expression in various *H. pylori* isolates indicated that rUreB is a good antigen candidate for developing *H. pylori* diagnostic tests and vaccines [26]. Furthermore, combinations of purified rUreA/rUreB have been shown to induce *H. pylori*-specific responses in human volunteers [27].

The *ureG* gene is a member of the *ure* gene cluster, and its encoded protein is expressed as an accessory protein required for nickel ion insertion into the apoenzyme of urease [26, 28]. In the biosynthesis process of the active metal-bound form of urease (a nickel-dependent enzyme), a lysine carbamate functional group is formed alongside the delivery of two Ni ^(2+)^ ions into the precast active site of the apoenzyme [15, 29]. UreG plays the role of chaperone in the urease active site assembly and is required to complete the biosynthesis of the nickel enzyme [15]. However, prior to our last report [14], UreG has not been described in terms of its potential as a diagnostic marker of *H. pylori* infection.

In the present study, rUreG was produced as both a soluble and insoluble protein, thus purification was performed under both native and denaturing conditions; however, the yield of the latter was almost twice that of the former. On the IgG and IgA western blot analysis, the reactivities of soluble rUreG were 70% and 60%, respectively, with sera from culture-positive patients. A combination of the IgG and IgA western blots gave a higher rate of reactivity of 83.3% (25/30). In comparison, in our previous study, the native UreG protein showed 73.3% (22/30) and 63.3% (19/30) reactivities when probed with IgG and IgA, respectively; while a combination of the IgG and IgA western blots showed 87% reactivity [14]. Thus, the diagnostic sensitivities of the native and recombinant forms of the UreG protein were similar. The specificity of the rUreG was found to be 97.5% and 92.5% when probed with anti-human IgG-HRP and anti-human IgA-HRP, respectively. In comparison, native UreG showed 100% specificity [14]. The reason for the higher specificity of native UreG compared with rUreG is not known. Mapping the epitope of the recombinant protein or the use of a peptide-based assay may lead to increased specificity of the recombinant protein.

Studies with urease showed that it yielded protection in only about 80% of immunized mice [30]. In a *Helicobacter felis* model, oral immunization in mice was performed with a combination of recombinant *H. pylori* heat-shock protein A (HspA) and urease; this dual antigen immunization induced 100% protection against *H. pylori* infection [31, 32]. This indicated that the combination of multiple antigens can be effective in the development of a good protective vaccine against *H. pylori* infection [30, 31]. Similarly, in another study, immunization with rHpaA or rUreB alone was found to induce weak immune responses; however, when both antigens were pooled, together with cholera toxin, strong immune responses were produced that induced protection against *H. pylori* infection [32, 33]. Because, in this study, rUreG has been shown to be immunogenic in mice, it should also be investigated for its potential as a vaccine candidate.

Similar to the multiple antigen concept mentioned above, in some commercial diagnostic kits, a mixture of antigens is used because it provides higher sensitivity and specificity compared with using a single antigen [17, 34–37]. This approach is further justified by reported findings that the sensitivity of some diagnostic kits varied depending on geographical location, with possible reasons being *H. pylori* strain heterogeneity, cross-reactivity with other intestinal pathogens, and varying immunological responses to *H. pylori* antigens in different patient populations [38–40]. In this study, although rUreG showed good seroreactivity to *H. pylori* patients’ sera, it did not show sufficiently high sensitivity to be used as a single antigen. Therefore, rUreG in combination with other potential diagnostic markers, such as rCagA, rUreB, and/or rVacA, may be investigated as a cocktail diagnostic reagent for the development of a robust test for *H. pylori* infection with effective worldwide application. Future studies should also be performed to evaluate the usefulness of rUreG in distinguishing the different clinical presentations of *H. pylori* infection, i.e., acute symptoms (such as duodenal ulcer or gastric cancer), chronic symptoms (such as dyspepsia), or no symptoms. The development of an ELISA using rUreG would be the preferred method for an effective diagnostic assay, however, further work to increase the purity of the recombinant protein may be required to maintain its high diagnostic specificity.

## Conclusion

In this study, the full-length, open reading frame of the 25-kDa UreG protein of *H. pylori*, a potential diagnostic marker, was cloned into the TOPO-TA vector, and sub-cloned into the pRSET expression vector. The recombinant form of the UreG protein showed high reactivity with *H. pylori* patients’ sera and no reactivity with most other sera, as well as good immunogenicity in mice. Thus, rUreG is a promising candidate for the diagnosis of *H. pylori* infection, either singly, or in combination with other diagnostic reagents.
